# Rapid lateral flow test for *Mycobacterium tuberculosis* complex and non-tuberculous mycobacteria differentiation

**DOI:** 10.1007/s00253-024-13293-1

**Published:** 2024-09-02

**Authors:** Ponrut Phunpae, Weeraya Thongkum, Wutthichai Panyasit, Witida Laopajon, Nuchjira Takheaw, Supansa Pata, Umpa Yasamut, Watchara Kasinrerk, Chatchai Tayapiwatana

**Affiliations:** 1 Division of Clinical Microbiology, Department of Medical Technology, Faculty of Associated Medical Sciences, Chiang Mai University, Chiang Mai, 50200 Thailand; 2 Division of Clinical Immunology, Department of Medical Technology, Faculty of Associated Medical Sciences, Chiang Mai University, Chiang Mai, 50200 Thailand; 3 Center of Innovative Immunodiagnostic Development, Department of Medical Technology, Faculty of Associated Medical Sciences, Chiang Mai University, Chiang Mai, 50200 Thailand; 4 Office of Disease Prevention and Control Region 1, Chiang Mai, 50100 Thailand; 5 Biomedical Technology Research Center, National Center for Genetic Engineering and Biotechnology, National Science and Technology Development Agency at the Faculty of Associated Medical Sciences, Chiang Mai University, Chiang Mai, 50200 Thailand

**Keywords:** *Mycobacterium tuberculosis* complex, Non-tuberculous mycobacteria, Ag85 protein, Immunochromatographic strip tests

## Abstract

**Abstract:**

The diagnosis of mycobacterial infections, including both the *Mycobacterium tuberculosis* complex (MTBC) and non-tuberculous mycobacteria (NTM), poses a significant global medical challenge. This study proposes a novel approach using immunochromatographic (IC) strip tests for the simultaneous detection of MTBC and NTM. Traditional methods for identifying mycobacteria, such as culture techniques, are hindered by delays in distinguishing between MTBC and NTM, which can affect patient care and disease control. Molecular methods, while sensitive, are resource-intensive and unable to differentiate between live and dead bacteria. In this research, we developed unique monoclonal antibodies (mAbs) against Ag85B, a mycobacterial secretory protein, and successfully implemented IC strip tests named 8B and 9B. These strips demonstrated high concordance rates with conventional methods for detecting MTBC, with positivity rates of 93.9% and 85.9%, respectively. For NTM detection, the IC strip tests achieved a 63.2% detection rate compared to culture methods, considering variations in growth rates among different NTM species. Furthermore, this study highlights a significant finding regarding the potential of MPT64 and Ag85B proteins as markers for MTBC detection. In conclusion, our breakthrough method enables rapid and accurate detection of both MTBC and NTM bacteria within the BACTEC MGIT system. This approach represents a valuable tool in clinical settings for distinguishing between MTBC and NTM infections, thereby enhancing the management and control of mycobacterial diseases.

**Key points:**

*• Panel of mAbs for differentiating MTB versus NTM*

*• IC strips for diagnosing MTBC and NTM after the BACTEC MGIT*

*• Combined detection of MTP64 and Ag85B enhances diagnostic accuracy*

**Supplementary Information:**

The online version contains supplementary material available at 10.1007/s00253-024-13293-1.

## Introduction

Tuberculosis (TB) remains as a persistent communicable ailment, posing a significant global public health challenge. Current estimates indicate that approximately one-fourth of the world's population is infected with MTBC (World Health Organization 2023). This disease is attributed to infection by the *Mycobacterium tuberculosis* complex (MTBC), with *M. tuberculosis* being the predominant causative agent (Sriruan et al. 2022). Only a fraction of infected individuals progress to active TB, with the majority remaining in the latent stage. Latent tuberculosis infection currently affects a quarter of the global population (Godoy 2021). In addition, infections caused by non-tuberculous mycobacteria (NTM) persist worldwide. NTM comprises about 190 species (Daley et al. 2020). Infection with NTM is commonly from the environment (Bryant et al. 2016). Most NTM infections in humans affect the lungs and differ in their growth rates. *Mycobacterium avium* complex (MAC), belonging to the slow-growing category, is the most common culprit of NTM. Additionally, *Mycobacterium abscessus* complex, categorized as a rapid grower (Park and Olivier 2015; Johnson and Odell 2014), contributes significantly to NTM lung disease. NTM can cause serious diseases in both healthy and immunocompromised individuals, resulting in significant illness and death (Pennington et al. 2021). Distinguishing between these infections is critical as they require distinct treatment approaches (Kim et al. 2021). The management of diseases caused by NTM is more complex than TB treatment. NTM strains exhibit resistance to most first-line antibiotics commonly used in TB therapy (Gopalaswamy et al. 2020; Griffith et al. 2007; Koh et al. 2005).

Accurate and swift diagnosis of TB is paramount for effective treatment and disease control. However, conventional diagnostic methods such as chest X-ray, tuberculin skin test, and acid-fast bacilli (AFB) staining of sputum exhibit low sensitivity and specificity, leading to potential misdiagnoses. Culture in liquid or solid media currently holds the gold standard for TB diagnosis. However, this method is time-consuming, taking 6–8 weeks for results, and required personal expert (Kashyap et al. 2007; Negi et al. 2005). The AFB smear microscopic method, therefore, still widely be accomplished in routine laboratory, especially in resource-limited countries due to its cost-effectiveness and ability to perform (Lewinsohn et al. 2017). During the last decades, culture of mycobacteria in liquid media is generally carried out using semi-automated the Mycobacteria Growth Indicator Tube (MGIT) 960 system, which takes ten days on average to detect mycobacterial growth (Caulfield and Wengenack 2016). Nevertheless, only mycobacterial growth can be detected in the MGIT system, and the species of mycobacteria cannot be identified.

Currently, several molecular techniques have been developed. The identification of microorganisms using nucleic acid amplification methods can significantly reduce the time required for TB diagnosis compared with traditional culture methods (Moore et al. 2005). The common molecular method is the Xpert MTB/RIF system, which makes specific TB diagnoses from sputum samples and can also detect drugs resistant to rifampicin (Gopalaswamy et al. 2020). However, this method exhibits lower sensitivity compared to the sputum-mycobacterial culture method (Shi et al. 2018). Importantly, it is unable to detect non-tuberculous mycobacteria (NTM) infections (Anand and Biswas 2021). Line Probe Assays are another molecular biology technique, including PCR to amplify the target DNA of the mycobacteria from the sample and then hybridize with a probe containing strips to capture the PCR product to identify mycobacteria (Gopalaswamy et al. 2020). Although molecular methods offer high specificity, it also has disadvantages, including complex procedures, necessitating expert handling, involving prohibitive costs, technical problems, and the requirement for specialized facilities (Machado et al. 2019). The molecular techniques can detect DNA from both live and dead bacteria, they may not be suitable for directly monitoring disease treatment in all cases (Garg et al. 2022).

Nowadays, rapid immunochromatographic tests (ICTs) based on a predominant protein MPT64 secreted by MTBC (Kumar et al. 2014) have been developed. ICTs provide quick results; hence, WHO recommended using ICTs for species identification in positive cultures (Kumar et al. 2014). The IC strip test for TB diagnosis is widely used in developing countries (Nurwidya et al. 2018). In addition to MPT64, antigen (Ag) 85 complex, a major secretory proteins of *M. tuberculosis*, was suggested to be a biomarker for TB diagnosis (Wiker and Harboe 1992; Kashyap et al. 2007). In our previous studies, we have generated three mAbs specifically targeting the Ag85B protein. The mAb clone AM85B-8 was characterized to react with Ag85B protein secreted by *Mycobacterium* spp. Afterward, by this mAb, an enzyme-linked immunosorbent assay (ELISA) was established for the detection of Ag85B protein in TB diagnosis (Phunpae et al. 2014). Subsequently, using a bio-layer interferometry, all produced monoclonal antibodies (mAbs) were characterized. It was found that mAb AM85B-5 and AM85B-8 reacted with Ag85B from various *Mycobacterium* species, while mAb AM85B-9 specifically targeted Ag85B of the MTBC (Chuensirikulchai et al. 2019). Although the antibody-biosensor system for detecting the Ag85B protein was successfully established to discriminate between MTBC and NTM, this method has not been validated with clinical specimens. We recognized that our ELISA and biosensor designs were too complex for routine clinical use. A rapid and user-friendly method is essential for general laboratory application.

In this study, we advanced our research by utilizing the generated mAbs to develop IC strip tests for diagnosing tuberculosis and distinguishing between MTBC and NTM. Leveraging the specificity of these mAbs, the IC strip tests offer a rapid and user-friendly platform, providing results in a significantly shorter time frame compared to standard mycobacterial methods.

## Materials and methods

### Production of large-scale antigen 85-specific monoclonal antibodies

Three hybridoma cell clones producing anti-Ag85B monoclonal antibodies (mAbs), namely AM85B-5 (Isotype IgG2b), AM85B-8 (IgG1), and AM85B-9 (IgG1), were generated in our laboratory (Phunpae et al. 2014; Chuensirikulchai et al. 2019). Large-scale production of anti-Ag85B mAbs from each clone involved cultivating hybridoma cells in serum-free media (Gibco, Grand Island, NY, USA) and purifying the antibodies through affinity chromatography using a protein L Sepharose column (GE Healthcare Bio-Sciences) (Phunpae et al. 2014). Purity evaluation of the purified mAbs was conducted using sodium dodecyl sulfate–polyacrylamide gel electrophoresis (SDS-PAGE).

### Determination of anti-Ag85B mAbs activity by enzyme-linked immunosorbent assay (ELISA)

Recombinant Ag85B and CD147 linked with biotin carboxyl carrier protein (BCCP) were synthesized as previously described (Phunpae et al. 2014; Tragoolpua et al. 2008). ELISA wells were coated with Ag85B-BCCP and CD147-BCCP as the irrelevant antigen control. After blocking, purified anti-Ag85B mAbs from each clone were added, and the antigen–antibody complexes were monitored using HRP-conjugated anti-mouse IgG. The absorbance was measured at 450 nm.

### Production of antibody conjugates for IC strip test

Colloidal gold conjugated with anti-Ag85B mAb clone AM85B-5 (AM85B-5-CGC) was synthesized according to Turkevich's method (Turkevich et al. 1951). The optimal antibody concentration for colloidal gold conjugation was determined by assessing absorption spectra. To validate the binding activity of AM85B-5-CGC, a dot blot immunoassay was conducted. Nitrocellulose membranes were dotted with recombinant Ag85B-BCCP, interferon gamma (IFN-γ), PBS, and goat anti-mouse Igs (KPL, Gaithersburg, MD, USA). Specific binding was assessed by probing with AM85B-5-CGC.

### Development of IC strip tests

**Fig. 1:**
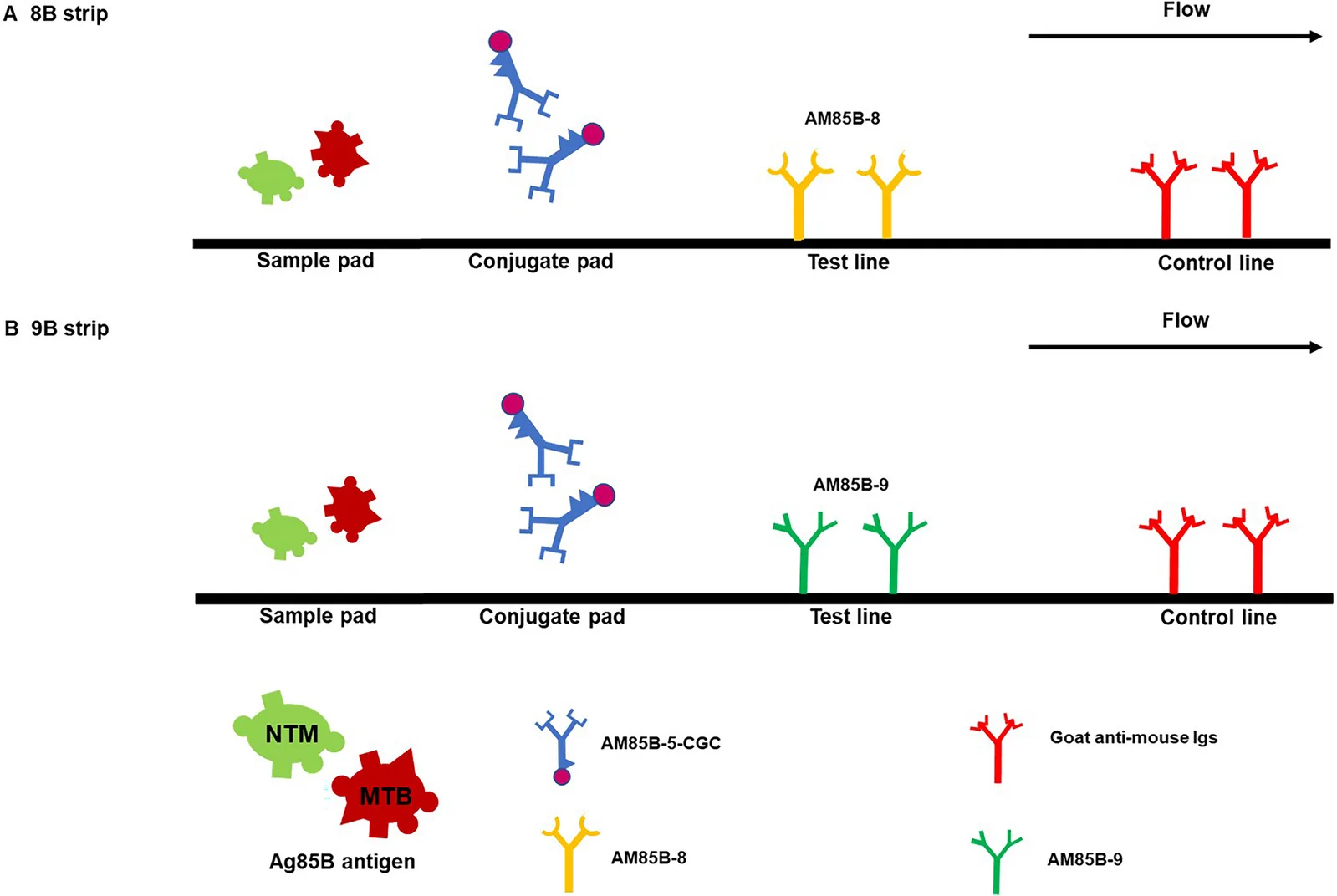
The essential elements of immunochromatographic strip tests with monoclonal antibodies targeting Ag85B protein for differentiation of MTBC and NTM. The anti-Ag85B mAb clone AM85B-5 conjugated with colloidal gold (AM85B-5-CGC) is applied on the conjugated pad of the nitrocellulose membrane strip. The AM85B-8 is immobilized at the test line in this IC strip test format, namely IC strip 8B (**A**). In the second strip, the AM85B-9 mAb is immobilized at the test line, this IC strip test pattern, namely IC strip 9B (**B**). In order to detect mycobacteria (MTBC and NTM), the sample pad is immersed in a liquid culture media containing mycobacterial Ag85B. If Ag85B is present, it will form immune complex with AM85B-5-CGC at the conjugate pad and moves to the test line area coated with AM85B-8 or AM85B-9 showing the red–purple line. Subsequently, the rest of AM85B-5-CGC moves upward to bind to goat anti-mouse Igs at the control line, producing the second red bands

IC strip tests for *Mycobacterium tuberculosis* complex (MTBC) and non-tuberculous mycobacteria (NTM) were developed using AM85B-5-CGC. The AM85B-8 or AM85B-9 was separately immobilized in the test line of IC strip test 8B for MTBC and NTM detection or IC strip test 9B for MTBC detection, respectively The AM85B-5-CGC was applied on the conjugated pad in both types of strips. The control line was immobilized with goat anti-mouse Igs. After immersing the samples, the results were read 10 min later. The essential elements of the IC strip test are depicted in Fig. 1.

### Clinical sample

Sputum samples submitted to the Tuberculosis Laboratory at the Office of Disease Prevention and Control Region 1 in Chiang Mai, Thailand for mycobacterial culture were utilized in this study.

### Mycobacterial culture in liquid media and solid media

The sputum specimens were decontaminated by using the NALC–NaOH method (Gopinath and Singh 2009) and processed for mycobacterial culture in the BACTEC MGIT 960 Mycobacterial Detection System (Becton Dickinson Diagnostic Instrument Systems, Sparks, MD, USA). Sample with positive signals was confirmed by AFB staining. The liquid cultures were subcultured on solid media for further identification of the presence of mycobacteria according to the standard method for mycobacterial culture. The MTBC from NTM were discriminated using routine clinical laboratory method. Briefly, mycobacteria suspension was inoculated onto the p-nitro benzoic acid test (PNB) containing L-J medium inhibiting MTBC (Kumar et al. 2014) and plain L-J medium. Then, incubate at 37 °C for 28 days. The PNB inhibition test differentiates MTBC from NTM by selectively inhibiting the growth of MTBC on culture medium containing PNB. NTM, being resistant to PNB, remains unaffected and continue to grow (Gopalaswamy et al. 2020).

### Evaluation of IC strip tests for tuberculosis diagnosis and MTBC/NTM differentiation

IC strip tests, specifically 8B and 9B, were utilized to diagnose tuberculosis and distinguish between MTBC and NTM infections in liquid culture media derived from positive MGIT tubes. The strips were immersed in the culture media, and observations of the lines at the test line and control line were made after 10 min. A positive result from strip 8B indicated the presence of MTBC or NTM. Positive results from strip 9B indicated the presence of MTBC, while negative results suggested the presence of NTM. The interpretation of the IC strip tests for detecting MTB and NTM is detailed in Figs. 6 and 7. Additionally, the SD BIOLINE TB Ag MPT64 Rapid assay (Abbott Inc, Yongin, South Korea) was employed concurrently for comparison with the developed strips. The effectiveness of these strips was further assessed in relation to the standard culture method described above.

## Results

### Anti-Ag85B monoclonal antibody and activity of anti-Ag85B mAb

**Fig. 2:**
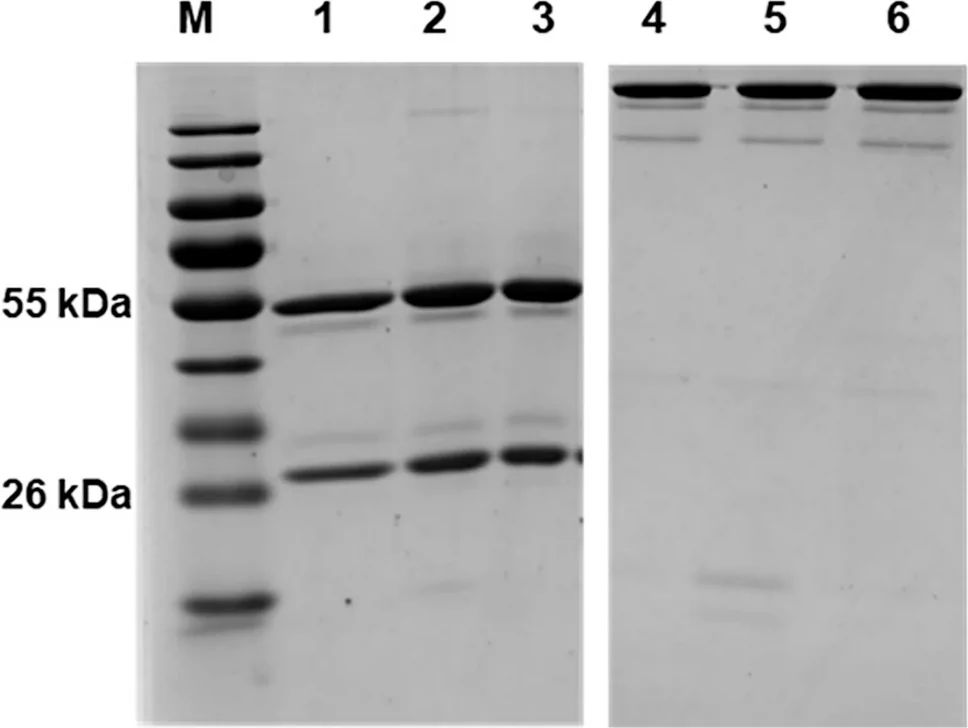
The anti-Ag85B mAbs purity tested by SDS-PAGE. The purified anti-Ag85 mAbs clone AM85B-5, AM85B-8, and AM85B-9 were examined for their purity by SDS-PAGE under reducing (lane 1–3) and non-reducing conditions (lane 4–6). The gels were then stained with Coomassie blue

**Fig. 3:**
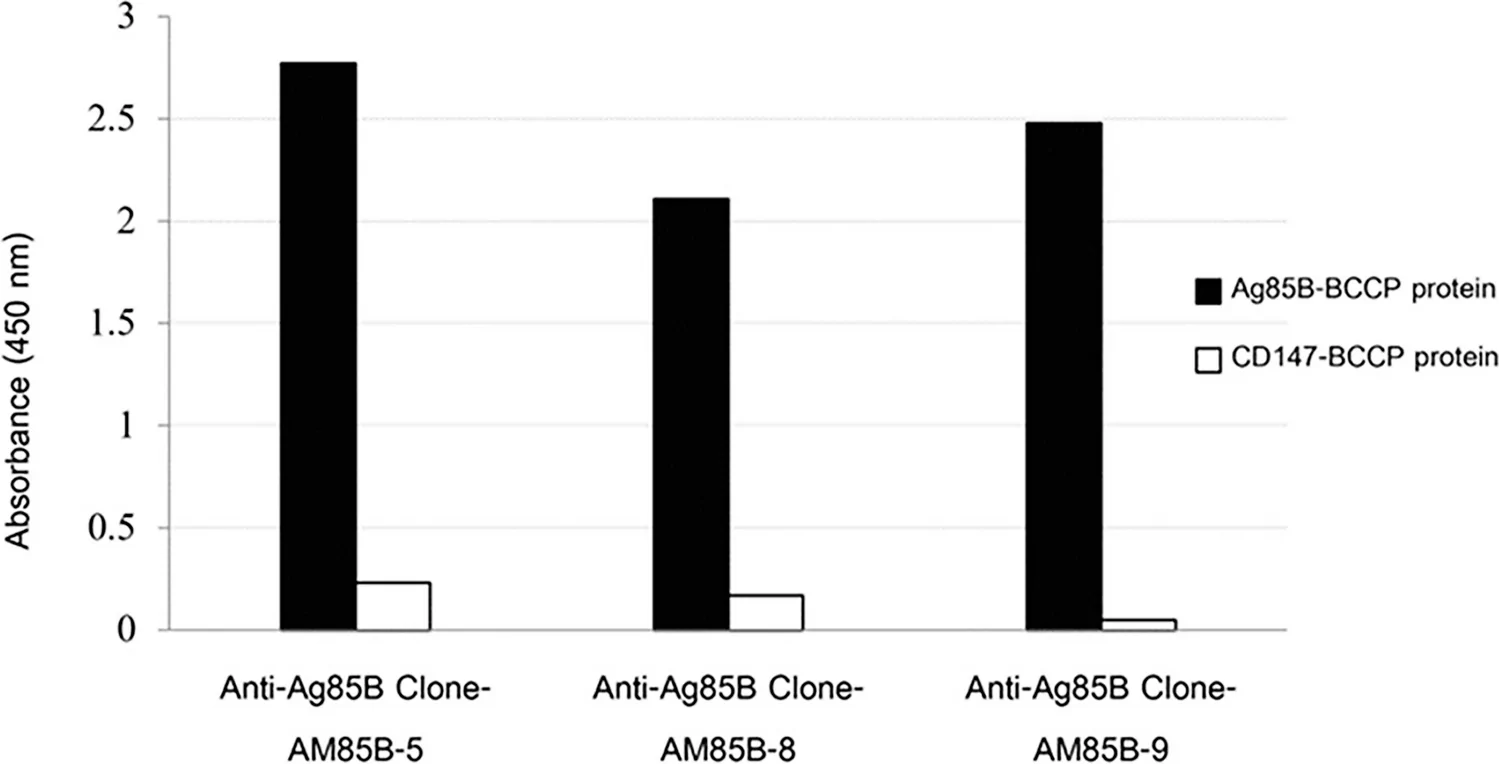
Verification of the reactivity of anti-Ag85 mAbs by indirect ELISA. Recombinant Ag85B-BCCP and CD147-BCCP proteins (control) were coated on the ELISA plate. Anti-Ag85B mAb clones AM85B-5, AM85B-8, and AM85B-9 were tested for their reactivity with Ag85B-BCCP and CD147-BCCP. The antigen–antibody complexes were detected using HRP-conjugated anti-mouse IgG. Absorbance was measured at 450 nm

The purity of anti-Ag85B clones AM85B-5, AM85B-8, and AM85B-9 was assessed through SDS-PAGE. Under reducing condition in SDS-PAGE, two protein bands at 50 and 25 kDa sizes were detected, corresponding to the heavy and light chains of the IgG antibody. Whereas a protein band at approximately 150 kDa was observed under nonreducing condition. Very few other contaminant proteins were detected, indicating high purity for each antibody clone (Fig. 2). The binding ability of the prepared anti-Ag85B mAbs was evaluated using ELISA. All three antibodies demonstrated significant binding to the specific antigen, Ag85B-BCCP. A higher absorbance value was observed when the antibodies reacted with Ag85B-BCCP, confirming their specific antigen-binding capability (Fig. 3).

### Development of an IC strip test for detection of Ag85B from mycobacteria

**Fig. 4:**
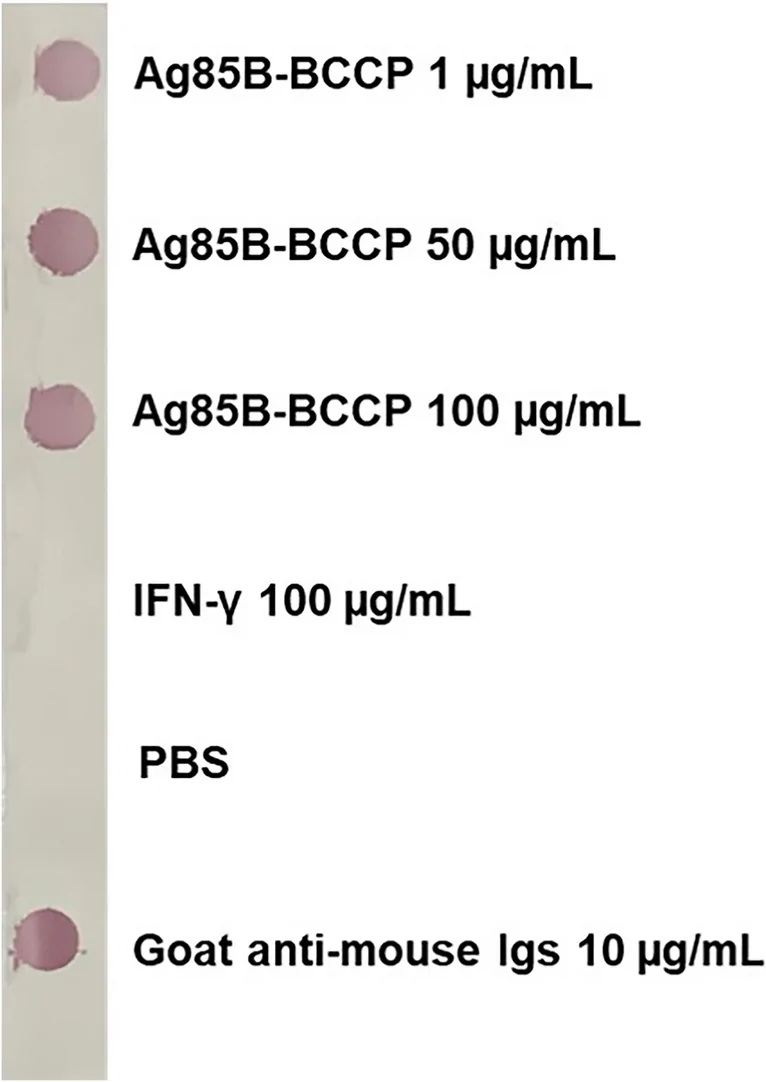
Testing the binding ability of anti-Ag85B mAb clone AM85B-5 conjugated with colloidal gold (AM85B-5-CGC) by direct dot immunoblotting assay. The nitrocellulose membrane was dotted with purified Ag85B-BCCP (at concentrations of 1, 50, and 100 µg/mL), IFN-γ (at concentrations of 100 µg/mL), PBS, and 10 µg/mL of goat anti-mouse Igs. Membrane was blocked by incubating with 5% (w/v) BSA in PBS. The AM85B-5-CGC was added to the dotted membrane at room temperature. Red–purple color-dots occurred, indicating the binding of AM85B-5-CGC to coated protein on membrane

**Fig. 5:**
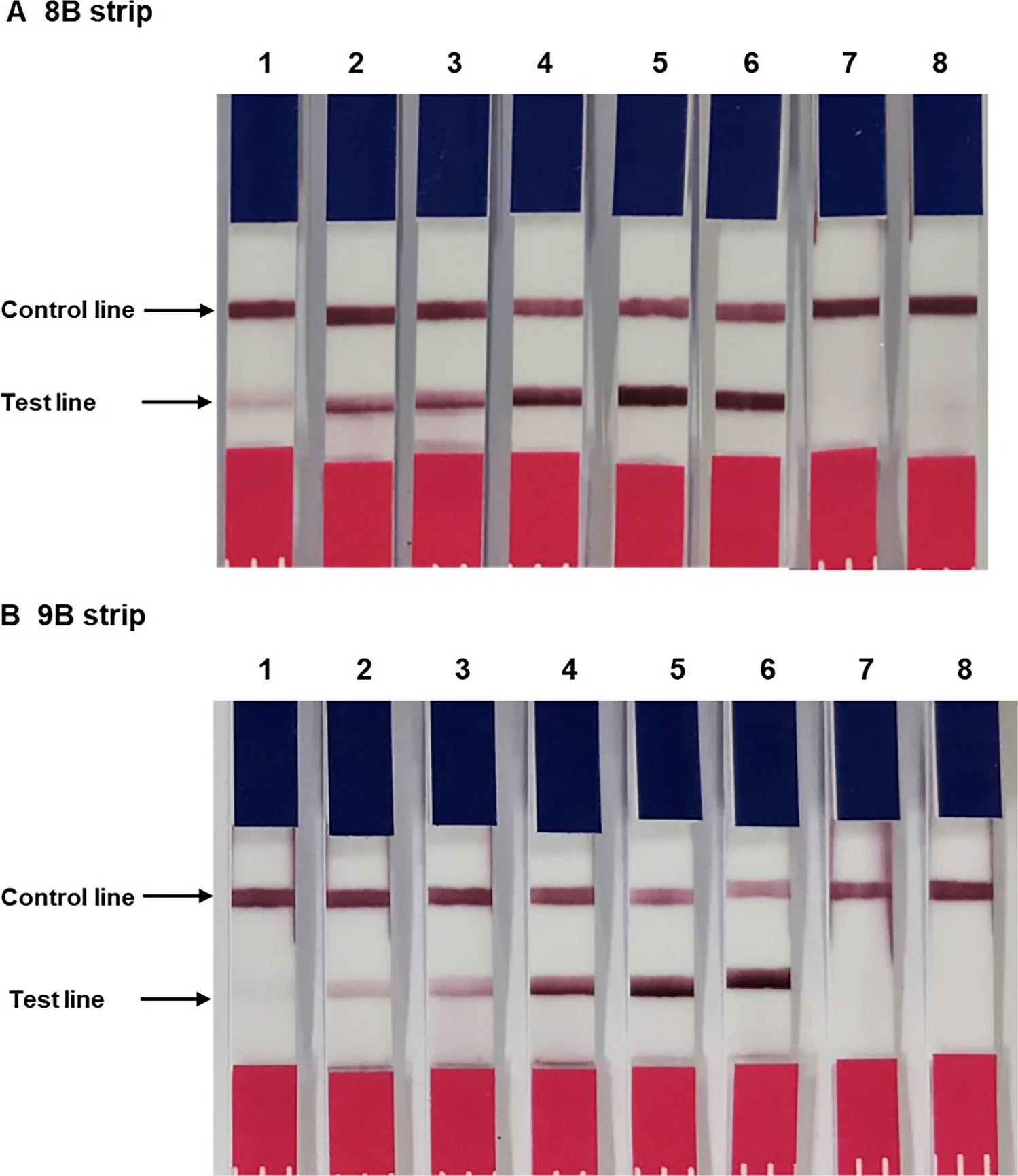
Evaluation of IC strips 8B and 9B for detecting mycobacterial Ag85B protein. The strip was created by immobilizing with AM85B-8 on the test line, namely strip 8B (**A**), and AM85B-9 on the test line, namely strip 9B (**B**). AM85B-5-CGC was sprayed on the conjugate pad of both strips. The sample pad of the assembled strips was immersed into Ag85B-BCCP suspended in MGIT liquid media at concentrations 0.01, 0.05, 0.1, 1, 5, and 10 µg/mL (strips no.1–6, respectively). IC strips no. 7 and 8 were tested with IFN- γ and MGIT media as a negative control, respectively. Strip 8B and 9B, no 1–6, show a positive reaction at the test and control lines. IC strips 8B and 9B, as well as strips no. 7 and 8, were immersed in IFN-γ and plain MGIT liquid media. No reaction was observed at the test line, while a positive reaction was evident at the control line

Anti-Ag85B clone AM85B-5 was labeled with colloidal gold and assessed through direct dot immunoblotting. The labeled antibodies demonstrated efficient binding with Ag85B, displaying no reactivity with irrelevant antigen, i.e., IFN-γ (Fig. 4). IC strips designed for tuberculosis diagnosis, capable of differentiating between MTBC and NTM based on the dot blot assay principle, were developed. In IC strip tests 8B and 9B, AM85B-5-CGC was applied to the conjugate pad of both strip types. For IC strip test 8B, AM85B-8 and goat anti-mouse Igs were immobilized at the test line and control line, respectively. In IC strip test 9B, a similar design was followed, but AM85B-9 was immobilized at the test line. The developed IC strip tests were evaluated against recombinant Ag85B protein at various concentrations, and both strips exhibited positive results consistently and proportionally (Fig. 5).

### Evaluation of IC strip test 8B and 9B for tuberculosis diagnosis and MTBC/NTM differentiation

**Fig. 6:**
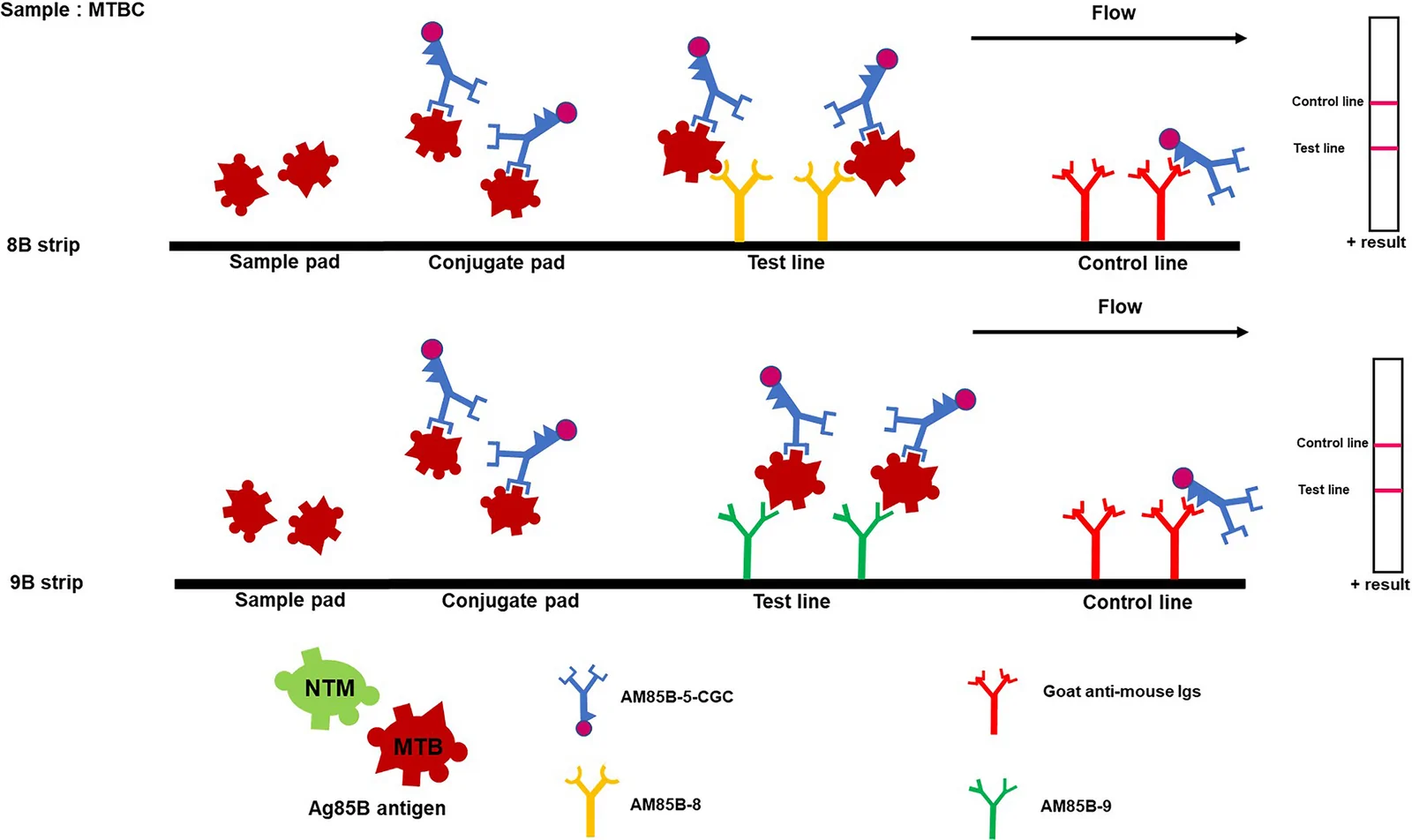
The IC strip principle and interpretation of the test results for identifying *Mycobacterium tuberculosis* complex (MTBC). This figure shows the principle of the IC strip test for the detection of Ag85B from MTBC in a liquid culture. A positive result is indicated by the presence of a red–purple color at both the test and control lines. Conversely, a red–purple color only at the control line signifies a negative result. When conducting tests for Ag85B secretion from MTBC, positive reactions were observed in both IC strips 8B and 9B

**Fig. 7:**
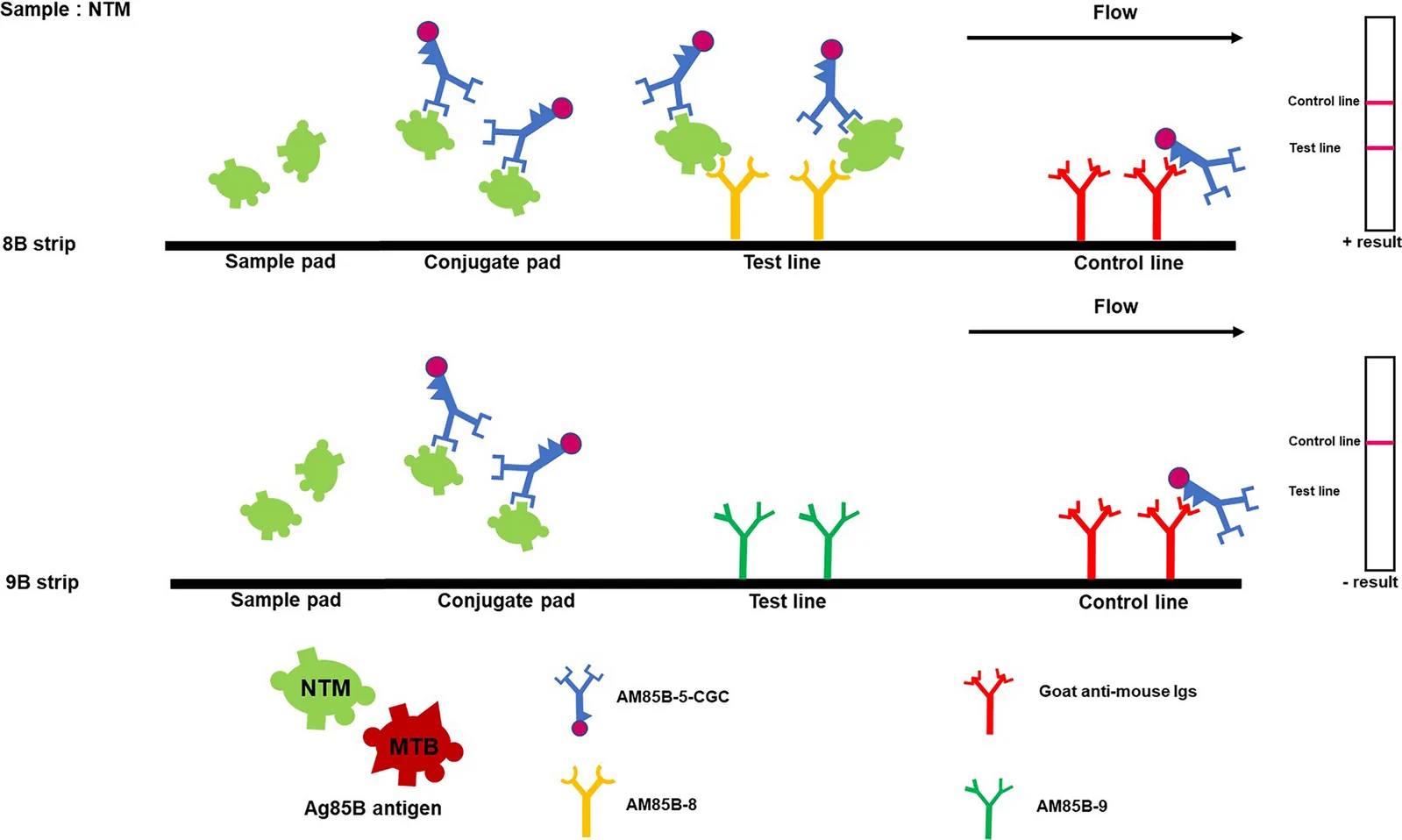
The IC strip and interpretation of the test results for identifying non-tuberculous mycobacteria (NTM). This figure shows the principle of the IC strip for Ag85B detection from NTM in a liquid culture. A red–purple color appearing at both the test and control lines indicates a positive result, while the presence of a red–purple color solely at the control line is indicative of a negative result. When testing for Ag85B secrete from NTM, IC strip 8B showed positive reaction, while strip 9B was negative reaction

The developed IC strip tests 8B and 9B were employed for detecting Ag85B from MTBC and NTM in liquid culture media from BACTEC MGIT 960 Mycobacterial Detection System. Positive readings were observed for MTBC and NTM, and the concurrent use of both strip tests enabled the differentiation between the two. Specifically, IC strip 8B provided positive results for both MTBC and NTM, while IC strip 9B yielded positive results only for MTBC (Figs. 6 and 7).

### Validating the IC strips with mycobacterial culture from sputum specimens

To evaluate TB diagnosis, IC strip tests 8B and 9B were employed to assess sputum culture in a liquid medium. Sputum samples were cultured in Mycobacteria Growth Indicator Tube (MGIT) and incubated in a BACTEC MGIT 960 Mycobacterial Detection System for monitoring bacterial growth. If bacterial growth occurred in a sample on any day, an alarm would be triggered. The culture media were then collected and tested using both IC strip tests 8B and 9B, as well as the SD BIOLINE TB Ag MPT64 strip test. Samples without alarms throughout the 42-day culture period were reported as “no growth” on day 42. Subsequently, the results from the strip tests were compared with the *Mycobacterium* results obtained from a standard culture system.

In this study, 129 specimens were analyzed, and *M. tuberculosis* was detected in 99 samples using the standard culture technique. Among the 99 specimens with the *M. tuberculosis* complex, 93.9% tested positive with IC strip test 8B, while 85.9% tested positive with IC strip test 9B. This indicates that the developed IC strip test 8B yielded consistent results with the diagnosis of *M. tuberculosis* infection by the culture method. Interestingly, the results from the commercially available IC strip test SD BIOLINE TB Ag MPT64 were only 86.9% consistent with the diagnosis of *M. tuberculosis* infection. When the 30 specimens indicating no *M. tuberculosis* growth were tested with IC strip tests 8B, 9B, and SD BIOLINE TB Ag MPT64 strip test (as shown in Table 1), 28 specimens (93%) tested negative with IC strip tests 8B and 9B, while 100% tested negative with SD BIOLINE TB Ag MPT64 strip test. These results suggest that the developed IC strip tests exhibit high consistency with the diagnosis of *M. tuberculosis* infection and negligible false positives (Table 1).

**Table 1:** Comparison of the specimens that tested positive for *M. tuberculosis* or not detected for *M. tuberculosis* by a standard mycobacterial culture system, along with detection of Ag85B with IC strip tests 8B, 9B, and SD BIOLINE TB Ag MPT64 test (MPT64)

No	IC strip test			Culture method identification
	MPT64	8B	9B	
1—82	+	+	+	MTB*
83—85	-	+	+	MTB*
86—89	+	+	-	MTB*
90—93	-	+	-	MTB*
94—99	-	-	-	MTB*
100—104	-	-	-	No Growth^**^
105	-	+	-	No Growth^**^
106—123	-	-	-	No Growth^**^
124	-	+	+	No Growth^**^
125–129	-	-	-	No Growth^**^

+ ; Positive result, -; Negative result

* When the MGIT tube showed a positive signal on any day

** Samples without alarms throughout the 42-day culture period were reported as no growth

A sputum sample was submitted for culture in mycobacteria growth indicator tube (MGIT) liquid culture systems

The liquid culture media was examined by IC strip test, 8B, 9B, and SD BIOLINE TB Ag MPT64

### Detection of NTM using the developed IC strip tests

To validate the ability in identifying NTM, IC strip tests 8B, and 9B were employed to test the culture medium of the 19 specimens confirmed for NTM infection. Specifically, specimens yielding culture results indicating NTM were tested positive with IC strip test 8B but negative with IC strip test 9B. As depicted in Table 2, the concurrent use of IC strip tests 8B and 9B successfully identified NTM infection in 12 specimens. The simultaneous use of both strip tests consistently provided accurate results for differentiating between MTBC and NTM infections. The IC strip tests 8B and 9B emerged as valuable tools for TB diagnosis, notably reducing diagnostic time compared to standard culture methods. IC strip test 8B detected *M. tuberculosis* infection in approximately 60% of specimens within 13 days post-culture, reaching 80% within 19 days post-culture. These findings highlight the potential for rapid and reliable TB screening using the developed IC strip test (Table 3).

**Table 2:** Comparison of the specimens detected for non-tuberculous mycobacteria (NTM) by a standard culture method, along with liquid culture testing with IC strip tests 8B and 9B

No	IC strip test		Culture method Identification
	8B	9B	
1—11	+	-	NTM
12	-	-	NTM
13	+	-	NTM
14	+	+	NTM
15—19	-	-	NTM

**Table 3:** Date of detection (days) when IC strip test 8B was used to test the specimens detected for *M. tuberculosis*, along with the number of specimens detected on that day (99 specimens in total)

Date of detection	Frequency of sample
7	4
8	1
9	6
10	17
11	10
12	7
13	14
14	3
15	3
16	5
17	3
18	4
19	4
21	5
23	1
24	1
26	2
27	1
28	1

## Discussion

The mycobacteria diagnosis, encompassing the *Mycobacterium tuberculosis* complex (MTBC) and non-tuberculous mycobacteria (NTM), poses a substantial global medical challenge. Significant pathogens within these groups, such as *M. tuberculosis* and NTM strains, including those in the *M. avium* complex, contribute significantly to disease burdens. Precise identification of mycobacteria is crucial for effective patient diagnosis, treatment monitoring, and disease prevention. The gold standard for tuberculosis diagnosis is the mycobacterial culture method, identifying live active bacteria species (Machado et al. 2019). Mycobacteria can be cultured using both liquid and solid media, with liquid media enabling faster growth (Moreira Ada et al. 2015). However, differentiating MTBC and NTM in positive liquid cultures remains a challenge, particularly in resource-limited countries with endemic TB. Facilities for identifying causative agents in these regions require improvement. Distinguishing between MTBC and NTM is critical for providing appropriate patient treatment (Kumar et al. 2014). Conventional methods using para-nitrobenzoic acid (PNB) for identification of MTBC and NTM, require pure isolates and lead to delayed results (Gopalaswamy et al. 2020). This delay impacts patient management and prolongs transmission among contacts, posing a significant limitation. Although, current molecular methods, such as the Xpert MTB/RIF system and line probe assays were developed. These techniques, however, are complex and resource-intensive. Their time-consuming nature remains a significant limitation. Despite their popularity for mycobacteria detection, molecular methods cannot differentiate between live and dead bacteria, necessitating intricate processes, specialized tools, and skilled personnel (Sibley et al. 2012)**.** These make the molecular method is unaffordable in resource-limited countries.

The MPT64 protein, specific to MTBC, and the Ag85 complex, predominantly Ag85B, serve as noteworthy secretory proteins detectable in liquid culture media, acting as valuable markers for MTBC detection (Orikiriza et al. 2017; Phunpae et al. 2014). Recent advancements in rapid MTBC identification from AFB-positive cultures rely on commercially available chromatographic detection of MPT64. Commonly available methods include the SD BIOLINE TB Ag MPT64 Rapid assay (Abbott Inc, Yongin, South Korea), the BD MGIT TBc identification test, and the Capilia TB-Neo assay (Tauns Laboratories, Inc., Numazu, Japan). These assays are user-friendly, and readily available, allowing results within 15 min from positive cultures, with high sensitivity and specificity (Gaillard et al. 2011). However, they are cost-effective and applicable only after significant growth of the bacteria in the medium which taking several days to weeks. In addition, MPT64 testing is limited due to its production and secretion by *M. tuberculosis*, *M. africanum*, and some *M. bovis* strains, and all members of the MTBC group. This protein's specificity for MTBC makes it unsuitable for directly detecting NTM infections, leading to potential false positives due to mutations in the coding region of the *MPT64* gene (Kumar et al. 2014; Singh et al. 2019).

Considering the Ag85B protein of mycobacteria, an in silico analysis in 2020 predicted B-cell epitopes, indicating its potential for diagnosis tests (Mufida et al. 2023; Karimah and Pambudi 2020). Variables in amino acid sequences of Ag85B proteins produced by various mycobacteria have been reported (Zhang et al. 2018). Previously, mAbs against Ag85B clones AM85B-5, AM85B-8 and AM85B-9, were successfully generated and characterized (Phunpae et al. 2014; Chuensirikulchai et al. 2019). Notably, mAbs AM85B-5 and AM85B-8 exhibited reactivity to Ag85B secreted by various *Mycobacterium* species, whereas mAb AM85B-9 specifically targeted Ag85B of the MTBC (Chuensirikulchai et al. 2019). To investigate the molecular basis for mAb AM85B-9 specificity, we performed amino acid sequence alignments of the Ag85 protein from diverse mycobacterial strains (Supplemental Fig. S1). This analysis identified a unique amino acid sequence, TQQIPK, at positions 234–239 of the MTB Ag85 protein, which differed significantly from the corresponding region in NTM strains. Further validation using the BCEP webserver to predict epitopes of the Ag85 structure (PDB 1F0N) identified TQQ_PK as a candidate epitope (Supplemental Fig. S2). Interestingly, the conserved amino acid I^236^ is not part of this epitope, which aligns with the observation that the I^236^ side chain is not exposed on the surface of Ag85. This suggests that TQQ_PK is a unique epitope accessible on the surface of Ag85 and specific to MTB. Epitope mapping experiments are ongoing to validate this observation and definitively identify the mAb-binding epitope.

Based on this information, mAbs AM85B-8 or AM85B-9 were immobilized on a nitrocellulose membrane to capture Ag85B proteins, facilitating subsequent binding of mAb AM85B-5-CGC. The efficacy of the developed IC strip tests (8B and 9B) was evaluated against MTBC culture results using standard methods. Results demonstrated high concordance, with IC strip test 8B yielding a 93.9% positivity rate, and IC strip test 9B showing 85.9% positivity, aligning with conventional culture methods. For NTM detection, the IC strip tests exhibited a 63.2% detection rate compared to the culture method, considering variations in growth rates and Ag85 secretion levels among different NTM species. While the study incubated bacteria at 37 °C (optimal for MTBC growth), further exploration may unveil nuances in NTM detection under varying conditions.

While the fabricated IC strips were directly utilized alongside the BACTEC MGIT system, certain considerations arise. For example, sputum samples that did not yield growth in the BACTEC MGIT system nevertheless tested positive with IC strip tests 8B and 9B (as shown in samples 105 and 124 in Table 1). This discrepancy could be attributed to the reported 1.93% false-negative rate of the BACTEC MGIT 960 system (Rishi et al. 2007), potentially affecting samples reported as having no growth in liquid culture. Furthermore, we propose enhancing the efficiency of MTBC detection by combining IC strip tests 8B and 9B with the MPT64 strip for testing samples. For instance, when MTB samples were tested, IC strip tests 8B and 9B yielded positive and negative results, respectively, whereas the MPT64 strip showed a positive result (sputum samples 86–89 in Table 1). In other examples, the MTB sample test with the MPT64 strip showed negative results, whereas testing with IC strips 8B and 9B revealed positive results (sputum samples 83–85 in Table 1). Combining Ag85 and MTP64 detection has the potential to improve MTB diagnosis by increasing sensitivity. The varying growth rates of different NTM species likely contribute to the discrepancy in NTM detection compared to culture methods (Kim et al. 2012). This variation can affect the secretion of Ag85B in different quantities. One instance observed in the study involved a positive result from the IC strip test 9B for an NTM sample (sample 14 in Table 2). This result could potentially be a false positive due to cross-reactions with other pathogens or a novel secretory protein not considered in the current study. However, by incorporating the MPT64 IC strip test alongside the 9B test, a more definitive diagnosis of NTM could be achieved. In some cases, a low amount of Ag85B may not be detected using IC strip test 8B, as observed in samples 12 and 15–19 in Table 1. Future studies could explore replacing CGC with liposome fluorescence labeling (Rink et al 2022) to potentially improve detection sensitivity.

This study introduces a novel method for tuberculosis testing, capable of detecting both MTBC and NTM using IC strips test 8B and 9B, a breakthrough not reported previously. The establishment of a rapid, one-step IC strip test utilizing mAbs specifically targeting the Ag85B protein stands as a pivotal achievement. Despite its significant contributions, this study has limitations. Direct sample sputum cannot be used due to its viscosity and lower Ag85B levels, restricting direct IC strip detection. However, the method effectively identifies the *M. tuberculosis* complex and NTM organisms within the BACTEC MGIT system. Future research may explore expanded applications and refine the testing process to enhance its clinical utility. This research marks a notable leap forward in tuberculosis diagnostics, presenting a swift, precise, and potentially cost-effective alternative to current methodologies.

## Supplementary Information

Below is the link to the electronic supplementary material.

Supplementary file1 (PDF 429 KB)

## Data Availability

All data generated or analyzed during the current study are available from the corresponding author upon reasonable request.

## References

[CR1] Anand AR, Biswas J (2021) TB or NTM: Can a new multiplex PCR assay be the answer? EBioMedicine 71:103552. 10.1016/j.ebiom.2021.103552 34455392 10.1016/j.ebiom.2021.103552 PMC8399081

[CR2] Bryant JM, Grogono DM, Rodriguez-Rincon D, Everall I, Brown KP, Moreno P, Verma D, Hill E, Drijkoningen J, Gilligan P, Esther CR, Noone PG, Giddings O, Bell SC, Thomson R, Wainwright CE, Coulter C, Pandey S, Wood ME, Stockwell RE, Ramsay KA, Sherrard LJ, Kidd TJ, Jabbour N, Johnson GR, Knibbs LD, Morawska L, Sly PD, Jones A, Bilton D, Laurenson I, Ruddy M, Bourke S, Bowler IC, Chapman SJ, Clayton A, Cullen M, Daniels T, Dempsey O, Denton M, Desai M, Drew RJ, Edenborough F, Evans J, Folb J, Humphrey H, Isalska B, Jensen-Fangel S, Jönsson B, Jones AM, Katzenstein TL, Lillebaek T, MacGregor G, Mayell S, Millar M, Modha D, Nash EF, O’Brien C, O’Brien D, Ohri C, Pao CS, Peckham D, Perrin F, Perry A, Pressler T, Prtak L, Qvist T, Robb A, Rodgers H, Schaffer K, Shafi N, van Ingen J, Walshaw M, Watson D, West N, Whitehouse J, Haworth CS, Harris SR, Ordway D, Parkhill J, Floto RA (2016) Emergence and spread of a human-transmissible multidrug-resistant nontuberculous *mycobacterium*. Science 354:751–757. 10.1126/science.aaf8156 27846606 10.1126/science.aaf8156 PMC5142603

[CR3] Caulfield AJ, Wengenack NL (2016) Diagnosis of active tuberculosis disease: From microscopy to molecular techniques. J Clin Tuberc Other Mycobact Dis 4:33–43. 10.1016/j.jctube.2016.05.005 31723686 10.1016/j.jctube.2016.05.005 PMC6850262

[CR4] Chuensirikulchai K, Laopajon W, Phunpae P, Apiratmateekul N, Surinkaew S, Tayapiwatana C, Pata S, Kasinrerk W (2019) Sandwich antibody-based biosensor system for identification of *Mycobacterium tuberculosis* complex and nontuberculous mycobacteria. J Immunoassay Immunochem 40(6):590–604. 10.1080/15321819.2019.1659814 31462139 10.1080/15321819.2019.1659814

[CR5] Daley CL, Iaccarino JM, Lange C, Cambau E, Wallace RJ Jr, Andrejak C, Böttger EC, Brozek J, Griffith DE, Guglielmetti L, Huitt GA (2020) Treatment of nontuberculous mycobacterial pulmonary disease: an official ATS/ERS/ESCMID/IDSA clinical practice guideline. Clin Infect Dis 71:e1–e36. 10.1093/cid/ciaa241 32628747 10.1093/cid/ciaa241 PMC7768748

[CR6] Gaillard T, Fabre M, Martinaud C, Vong R, Brisou P, Soler C (2011) Assessment of the SD Bioline Ag MPT64 Rapid™ and the MGIT™ TBc identification tests for the diagnosis of tuberculosis. Diagn Microbiol Infect Dis 70(1):154–156. 10.1016/j.diagmicrobio.2010.12.011 21397427 10.1016/j.diagmicrobio.2010.12.011

[CR7] Garg A, Agarwal L, Mathur R (2022) Role of GeneXpert or CBNAAT in diagnosing tuberculosis: Present scenario. Med J DY Patil Vidyapeeth 15(1):14–19. 10.4103/mjdrdypu.mjdrdypu_182_20

[CR8] Godoy P (2021) Guidelines on controlling latent tuberculosis infection to support tuberculosis elimination. Rev Esp Sanid Penit 23:28–36. 10.18176/resp.00028 33847703 10.18176/resp.00028 PMC8278168

[CR9] Gopalaswamy R, Shanmugam S, Mondal R, Subbian S (2020) Of tuberculosis and non-tuberculous mycobacterial infections–a comparative analysis of epidemiology, diagnosis and treatment. J Biomed Sci 27:74. 10.1186/s12929-020-00667-6 32552732 10.1186/s12929-020-00667-6 PMC7297667

[CR10] Gopinath K, Singh S (2009) Multiplex PCR assay for simultaneous detection and differentiation of *Mycobacterium tuberculosis*, *Mycobacterium avium* complexes and other Mycobacterial species directly from clinical specimens. J Appl Microbiol 107(2):425–435. 10.1111/j.1365-2672.2009.04218.x 19302308 10.1111/j.1365-2672.2009.04218.x

[CR11] Griffith DE, Aksamit T, Brown-Elliott BA, Catanzaro A, Daley C, Gordin F, Holland SM, Horsburgh R, Huitt G, Iademarco MF, Iseman M, Olivier K, Ruoss S, von Reyn CF, Wallace RJ Jr, Winthrop K (2007) ATS Mycobacterial Diseases Subcommittee; American Thoracic Society; Infectious Disease Society of America. An official ATS/IDSA statement: diagnosis, treatment, and prevention of nontuberculous mycobacterial diseases. Am J Respir Crit Care Med 175(4):367–416. 10.1164/rccm.200604-571ST 17277290 10.1164/rccm.200604-571ST

[CR12] Johnson MM, Odell JA (2014) Nontuberculous mycobacterial pulmonary infections. J Thorac Dis 6(3):210–220. 10.3978/j.issn.2072-1439.2013.12.24 24624285 10.3978/j.issn.2072-1439.2013.12.24 PMC3949190

[CR13] Karimah N, Pambudi S (2020) Prediction of B-cell epitope by in silico analysis of *Mycobacterium tuberculosis* Ag85B antigen. AsPac J Mol Biol Biotechnol 101–109. 10.35118/apjmbb.2020.028.1.10

[CR14] Kashyap RS, Rajan AN, Ramteke SS, Agrawal VS, Kelkar SS, Purohit HJ, Taori GM, Daginawala HF (2007) Diagnosis of tuberculosis in an Indian population by an indirect ELISA protocol based on detection of Antigen 85 complex: a prospective cohort study. BMC Infect Dis 7:74. 10.1186/1471-2334-7-74 17620147 10.1186/1471-2334-7-74 PMC1933431

[CR15] Kim CJ, Kim NH, Song KH, Choe PG, Kim ES, Park SW, Kim HB, Kim NJ, Kim EC, Park WB, Oh MD (2012) Differentiating rapid- and slow-growing mycobacteria by difference in time to growth detection in liquid media. Diagn Microbiol Infect Dis 75:73–6. 10.1016/j.diagmicrobio.2012.09.019 23114094 10.1016/j.diagmicrobio.2012.09.019

[CR16] Kim MJ, Kim KM, Shin JI, Ha JH, Lee DH, Choi JG, Park JS, Byun JH, Yoo JW, Eum S, Jung M, Baik SC, Lee WK, Kang HL, Shin MK (2021) Identification of nontuberculous mycobacteria in patients with pulmonary diseases in Gyeongnam, Korea, using multiplex PCR and multigene sequence-based analysis. Can J Infect Dis Med Microbiol 2021:8844306. 10.1155/2021/8844306 33688383 10.1155/2021/8844306 PMC7920741

[CR17] Koh WJ, Kwon OJ, Lee KS (2005) Diagnosis and treatment of nontuberculous mycobacterial pulmonary diseases: a Korean perspective. J Korean Med Sci 20(6):913–925. 10.3346/jkms.2005.20.6.913 16361797 10.3346/jkms.2005.20.6.913 PMC2779319

[CR18] Kumar P, Benny P, Jain M, Singh S (2014) Comparison of an in-house multiplex PCR with c differentiation of MTB from NTM isolates. Int J Mycobacteriol 3:50–56. 10.1016/j.ijmyco.2013.12.001 26786223 10.1016/j.ijmyco.2013.12.001

[CR19] Lewinsohn DM, Leonard MK, Mazurek AM, LoBue PA, Cohn DL, Daley CL, Desmond E, Keane J, Lewinsohn DA, Loeffler GH, O’Brien RJ, Pai M, Richeldi L, Salfinger M, Shinnick TM, Sterling TR, Warshauer DM, Woods GL (2017) Official American Thoracic Society/Infectious Diseases Society of America/Centers for Disease Control and Prevention Clinical Practice Guidelines: Diagnosis of Tuberculosis in Adults and Children. Clin Infect Dis 64:111–115. 10.1093/cid/ciw778 28052967 10.1093/cid/ciw778 PMC5504475

[CR20] Machado D, Couto I, Viveiros M (2019) Advances in the molecular diagnosis of tuberculosis: from probes to genomes. Infect Genet Evol 72:93–112. 10.1016/j.meegid.2018.11.021 30508687 10.1016/j.meegid.2018.11.021

[CR21] Moore DF, Guzman JA, Mikhail LT (2005) Reduction in turnaround time for laboratory diagnosis of pulmonary tuberculosis by routine use of a nucleic acid amplification test. Diagn Microbiol Infect Dis 52(3):247–254. 10.1016/j.diagmicrobio.2005.02.014 15893903 10.1016/j.diagmicrobio.2005.02.014

[CR22] Moreira Ada S, Huf G, Vieira MA, Costa PA, Aguiar F, Marsico AG, Fonseca Lde S, Ricks M, Oliveira MM, Detjen A, Fujiwara PI, Squire SB, Kritski AL (2015) Liquid vs solid culture medium to evaluate proportion and time to change in management of suspects of tuberculosis—a pragmatic randomized trial in secondary and tertiary health care units in Brazil. PLoS ONE 10(6):e0127588. 10.1371/journal.pone.0127588 26046532 10.1371/journal.pone.0127588 PMC4457845

[CR23] Mufida DC, Aziz AM, Misturiansyah NI (2023) Potential of B-cell epitopes protein Ag85 complex *Mycobacterium tuberculosis* as serodiagnostic antigen of tuberculosis by in silico study. JJBTR 9:7–13. 10.14710/jbtr.v9i1.16379

[CR24] Negi SS, Khan SF, Gupta S, Pasha ST, Khare S, Lal S (2005) Comparison of the conventional diagnostic modalities, bactec culture and polymerase chain reaction test for diagnosis of tuberculosis. Indian J Med Microbiol 23:29–33. 10.4103/0255-0857.13869 15928418 10.4103/0255-0857.13869

[CR25] Nurwidya F, Handayani D, Burhan E, Yunus F (2018) Molecular diagnosis of tuberculosis. Chonnam Med J 54:1–9. 10.4068/cmj.2018.54.1.1 29399559 10.4068/cmj.2018.54.1.1 PMC5794472

[CR26] Orikiriza P, Nyehangane D, Atwine D, Kisakye JJ, Kassaza K, Amumpaire JM, Boum Y 2nd (2017) Evaluation of the SD Bioline TB Ag MPT64 test for identification of *Mycobacterium tuberculosis* complex from liquid cultures in southwestern Uganda. Afr J Lab Med 6(2):383. 10.4102/ajlm.v6i2.383 28879157 10.4102/ajlm.v6i2.383 PMC5523908

[CR27] Park IK, Olivier KN (2015) Nontuberculous mycobacteria in cystic fibrosis and non-cystic fibrosis bronchiectasis. Semin Respir Crit Care Med 36:217–224. 10.1055/s-0035-1546751 25826589 10.1055/s-0035-1546751 PMC7171444

[CR28] Pennington KM, Vu A, Challener D, Rivera CG, Shweta FNU, Zeuli JD, Temesgen Z (2021) Approach to the diagnosis and treatment of non-tuberculous mycobacterial disease. J Clin Tuberc Other Mycobact Dis 24:100244. 10.1016/j.jctube.2021.100244 34036184 10.1016/j.jctube.2021.100244 PMC8135042

[CR29] Phunpae P, Chanwong S, Tayapiwatana C, Apiratmateekul N, Makeudom A, Kasinrerk W (2014) Rapid diagnosis of tuberculosis by identification of Antigen 85 in mycobacterial culture system. Diagn Microbiol Infect Dis 78(3):242–248. 10.1016/j.diagmicrobio.2013.11.028 24418370 10.1016/j.diagmicrobio.2013.11.028

[CR30] Rink S, Kaiser B, Steiner MS, Duerkop A, Baeumner AJ (2022) Highly sensitive interleukin 6 detection by employing commercially ready liposomes in an LFA format. Anal Bioanal Chem 414:3231–3241. 10.1007/s00216-021-03750-5 34773470 10.1007/s00216-021-03750-5 PMC8590136

[CR31] Rishi S, Sinha P, Malhotra B, Pal N (2007) A comparative study for the detection of Mycobacteria by BACTEC MGIT 960, Lowenstein Jensen media and direct AFB smear examination. Indian J Med Microbiol 25(4):383–386. 10.4103/0255-0857.37344 18087090 10.4103/0255-0857.37344

[CR32] Shi J, Dong W, Ma Y, Liang Q, Shang Y, Wang F, Huang H, Pang Y (2018) GeneXpert MTB/RIF outperforms mycobacterial culture in detecting *Mycobacterium tuberculosis* from salivary sputum. Biomed Res Int 2018:1514381. 10.1155/2018/1514381 29805972 10.1155/2018/1514381 PMC5899871

[CR33] Sibley CD, Peirano G, Church DL (2012) Molecular methods for pathogen and microbial community detection and characterization: current and potential application in diagnostic microbiology. Infect Genet Evol 12:505–521. 10.1016/j.meegid.2012.01.011 22342514 10.1016/j.meegid.2012.01.011 PMC7106020

[CR34] Singh K, Kumari R, Tripathi R, Gupta A, Anupurba S (2019) Mutation in *MPT64* gene influencing diagnostic accuracy of SD Bioline assay (capilia). BMC Infect Dis 19:1048. 10.1186/s12879-019-4671-2 31829183 10.1186/s12879-019-4671-2 PMC6907232

[CR35] Sriruan C, Kasinrerk W, Intorasoot S, Butr-Indr B, Netirat N, Khantipongse J, Phunpae P (2022) Identification and monitoring of *Mycobacterium tuberculosis* growth in liquid culture by antigen 85 detection. J Assoc Med Sci 55:96–104 (https://he01.tci-thaijo.org/index.php/bulletinAMS/article/view/255554)

[CR36] Tragoolpua K, Intasai N, Kasinrerk W, Mai S, Yuan Y, Tayapiwatana C (2008) Generation of functional scFv intrabody to abate the expression of CD147 surface molecule of 293A cells. BMC Biotechnol 8:5. 10.1186/1472-6750-8-5 18226275 10.1186/1472-6750-8-5 PMC2258298

[CR37] Turkevich J, Stevenson PC, Hillier J (1951) A study of the nucleation and growth processes in the synthesis of colloidal gold. Discuss Faraday Soc 11:55–75. 10.1039/DF9511100055

[CR38] Wiker HG, Harboe M (1992) The antigen 85 complex: a major secretion product of *Mycobacterium tuberculosis*. Microbiol Rev 56(4):648–661. 10.1128/mr.56.4.648-661.1992 1480113 10.1128/mr.56.4.648-661.1992 PMC372892

[CR39] World Health Organization (2023) Global tuberculosis report 2023. https://www.who.int/teams/global-tuberculosis-programme/tb-reports/global-tuberculosis-report-2023. Accessed 29 Jul 2024

[CR40] Zhang W, Shu Q, Zhao Z, Fan J, Lyon CJ, Zelazny AM, Hu Y (2018) Antigen 85B peptidomic analysis allows species-specific mycobacterial identification. Clin Proteomics 15:1. 10.1186/s12014-017-9177-6 29321721 10.1186/s12014-017-9177-6 PMC5757288

