# Supplementary material for: Rapid lateral flow test for Mycobacterium tuberculosis complex and non-tuberculous mycobacteria differentiation
Source: Appl Microbiol Biotechnol. 2024 Sep 2;108:456. doi: 10.1007/s00253-024-13293-1 (PMC11369055; doi:10.1007/s00253-024-13293-1)
Supplement: Supplementary file 1 [file 253_2024_13293_MOESM1_ESM.pdf]

Supplementary Information

Rapid lateral flow test for *Mycobacterium tuberculosis* complex and non-tuberculous mycobacteria differentiation

Ponrut Phunpae[1]*, Weeraya Thongkum[2, 3]*, Wutthichai Panyasit[4], Witida Laopajon[2, 5], Nuchjira Takheaw[2, 5], Supansa Pata[2, 5],Umpa Yasamut[2, 3],Watchara Kasinrerk[2, 5#],Chatchai Tayapiwatana[2, 3#]

1. Division of Clinical Microbiology, Department of Medical Technology, Faculty of Associated Medical Sciences, Chiang Mai University, Chiang Mai 50200, Thailand
2. Division of Clinical Immunology, Department of Medical Technology, Faculty of Associated Medical Sciences, Chiang Mai University, Chiang Mai 50200, Thailand
3. Center of Innovative Immunodiagnostic Development, Department of Medical Technology, Faculty of Associated Medical Sciences, Chiang Mai University, Chiang Mai 50200, Thailand
4. Office of Disease Prevention and Control Region 1, Chiang Mai 50100, Thailand
5. Biomedical Technology Research Center, National Center for Genetic Engineering and Biotechnology, National Science and Technology Development Agency at the Faculty of Associated Medical Sciences, Chiang Mai University, Chiang Mai 50200, Thailand

* Ponrut Phunpae and Weeraya Thongkum contributed equally to this work.

[#]**Corresponding authors:**

Watchara Kasinrerk.
Division of Clinical Immunology, Department of Medical Technology, Faculty of Associated Medical Sciences, Chiang Mai University, Chiang Mai 50200, Thailand
Telephone: +6653935070
E-mail: Watchara.k@cmu.ac.th;

Chatchai Tayapiwatana.
Division of Clinical Immunology, Department of Medical Technology, Faculty of Associated Medical Sciences, Chiang Mai University, Chiang Mai 50200, Thailand
Telephone: +66818845141
E-mail: asimi002@hotmail.com

```
P9WQP1|A85B_MYCTU    MTDVSRKIR-AWGRRLMIGTAAAVVLPGLVGLAGGAATAGAFSRPGLPVEYLQVPSPSMG 59
P0C2T2|A85B_MYCBO    MTDVSRKIR-AWGRRLMIGTAAAVVLPGLVGLAGGAATAGAFSRPGLPVEYLQVPSPSMG 59
Q49575|A85B_MYCIA    MTDLSEKVR-AWGRRLVVGAAAAATLPGLIGIAGGAATANAFSRPGLPVEYLQVPSAGMG 59
A1KJU9|A85B_MYCBP    MTDVSRKIR-AWGRRLMIGTAAAVVLPGLVGLAGGAATAGAFSRPGLPVEYLQVPSPSMG 59
P21160|A85B_MYCKA    MTDVSGKIR-AWGRRLLVGAAAAAALPGLVGLAGGAATAGAFSRPGLPVEYLQVPSAAMG 59
Q06947|A85B_MYCAV    MTDLSEKVR-AWGRRLLVGAAAAVTLPGLIGLAGGAATANAFSRPGLPVEYLQVPSAGMG 59
B1MMV2|B1MMV2_MYCA9  -MEFLQKMRGATARLAAVAAVAAAILPGLIGVTGGSAIAGAFSRPGLPVEYLQVPSPAMG 59
                       :.  *:* * .*   :.:.**. ****:*::**:* *.**************** .**

P9WQP1|A85B_MYCTU    RDIKVQFQSGGNNSPAVYLLDGLRAQDDYNGWDINTPAFEWYYQSGLSIVMPVGGQSSFY 119
P0C2T2|A85B_MYCBO    RDIKVQFQSGGNNSPAVYLLDGLRAQDDYNGWDINTPAFEWYYQSGLSIVMPVGGQSSFY 119
Q49575|A85B_MYCIA    RDIKVQFQSGGNGSPAVYLLDGLRAQDDYNGWDINTPAFEWYYQSGLSVIMPVGGQSSFY 119
A1KJU9|A85B_MYCBP    RDIKVQFQSGGNNSPAVYLLDGLRAQDDYNGWDINTPAFEWYYQSGLSIVMPVGGQSSFY 119
P21160|A85B_MYCKA    RSIKVQFQSGGDNSPAVYLLDGLRAQDDYNGWDINTPAFEWYYQSGLSVIMPVGGQSSFY 119
Q06947|A85B_MYCAV    RDIKVQFQSGGNGSPAVYLLDGLRAQDDYNGWDINTPAFEWYYQSGLSVIMPVGGQSSFY 119
B1MMV2|B1MMV2_MYCA9  RDIVVEFQPGGP--HAVYLLDGQRAREDYNGWDIETTAFEDYYQSGISVVMPVGGQSSNY 117
                     *.* *:** **    ******* **::*******:* *** *****:*::******** *

P9WQP1|A85B_MYCTU    SDWYSPACGKAGCQTYKWETFLTSELPQWLSANRAVKPTGSAAIGLSMAGSSAMILAAYH 179
P0C2T2|A85B_MYCBO    SDWYSPACGKAGCQTYKWETFLTSELPQWLSANRAVKPTGSAAIGLSMAGSSAMILAAYH 179
Q49575|A85B_MYCIA    ADWYQPACGKAGCSTYKWETFLTSELPQYLASNKGVKSTGSAAVGISMSGSSAMILAVNH 179
A1KJU9|A85B_MYCBP    SDWYSPACGKAGCQTYKWETLLTSELPQWLSANRAVKPTGSAAIGLSMAGSSAMILAAYH 179
P21160|A85B_MYCKA    SDWYSPACGKAGCTTYKWETFLTSELPQWLSANRSVKPTGSAAVGISMAGSSALILSVYH 179
Q06947|A85B_MYCAV    ADWYQPACGKAGCSTYKWETFLTSELPSYLASNKGVKRTGNAAVGISMSGSSAMILAVNH 179
B1MMV2|B1MMV2_MYCA9  TDWYNPAKGKDGVWTYKWETFLTTELPQWLGVNKGISPAANAVVGLSMAGPSALTLAIYH 177
                     :***.** ** *  ******:**:***.:*. *:.:. :..*.:*:**:* **: *:  *

P9WQP1|A85B_MYCTU    PQQFIYAGSLSALLDPSQGMGPSLIGLAMGDAGGYKAADMWGPSSDPAWERNDPTQQIPK 239
P0C2T2|A85B_MYCBO    PQQFIYAGSLSALLDPSQGMGPSLIGLAMGDAGGYKAADMWGPSSDPAWERNDPTQQIPK 239
Q49575|A85B_MYCIA    PNQFVYAGSLSALLDPSQGMGPSLIGLAMGDAGGYKADAMWGPSSDPAWQRNDPSLQIPA 239
A1KJU9|A85B_MYCBP    PQQFIYAGSLSALLDPSQGMGPSLIGLAMGDAGGYKAADMWGPSSDPAWERNDPTQQIPK 239
P21160|A85B_MYCKA    PQQFIYAGSLSALMDPSQGMGPSLIGLAMGDAGGYKASDMWGPSSDPAWQRNDPSLHIPE 239
Q06947|A85B_MYCAV    PDQFIYAGSLSALLDPSQGMGPSLIGLAMGDAGGYKADAMWGPSSDPAWQRNDPSLHIPE 239
B1MMV2|B1MMV2_MYCA9  PQQFVYAGALSAPLHPSDQK--WQIRVAMSDAGGFNADDMWGPDSDPAWARNDPFLHIDQ 235
                     *:**:***:*** :.**:      * :**.****::*  ****.***** ****  :*

P9WQP1|A85B_MYCTU    LVANNTRLWVYCGNGTPNELGGAN-----IPAEFLENFVRSSNLKFQDAYNAAGGHNAVF 294
P0C2T2|A85B_MYCBO    LVANNTRLWVYCGNGTPNELGGAN-----IPAEFLENFVRSSNLKFQDAYNAAGGHNAVF 294
Q49575|A85B_MYCIA    LVGNNTRLWVYCGNGTPSELGGAN-----MPAEFLENFVRSSNLKFQDAYNAAGGHNAVF 294
A1KJU9|A85B_MYCBP    LVANNTRLWVYCGNGTPNELGGAN-----IPAEFLENFVRSSNLKFQDAYNAAGGHNAVF 294
P21160|A85B_MYCKA    LVANNTRLWIYCGNGTPSELGGAN-----VPAEFLENFVRSSNLKFQDAYNAAGGHNAVF 294
Q06947|A85B_MYCAV    LVGHNTRLWLYCGNGTPSELGGAN-----MPAEFLENFVRSSNLKFQDAYNGAGGHNAVF 294
B1MMV2|B1MMV2_MYCA9  LIANNTRLWIYCGSGDATDLDKDRNGLEVISGGVIEGQVIDSDKKFAEAYGSAGGANAHF 295
                     *:.:*****:***.*  .:*.  .     : . .:*. * .*: ** :**..*** ** *

P9WQP1|A85B_MYCTU    NFPPNGTHSWEYWGAQLNAMKGDLQSSLGAG----- 325
P0C2T2|A85B_MYCBO    NFPPNGTHSWEYWGAQLNAMKGDLQSSLGAG----- 325
Q49575|A85B_MYCIA    NFNANGTHSWEYWGAQLNAMKPDLQSALGASSGGGG 330
A1KJU9|A85B_MYCBP    NFPPNGTHSWEYWGAQLNAMKGDLQSSLGAG----- 325
P21160|A85B_MYCKA    NLDANGTHSWEYWGAQLNAMKGDLQASLGAR----- 325
Q06947|A85B_MYCAV    NFNANGTHSWEYWGAQLNAMKPDLQGTLGASPGGGG 330
B1MMV2|B1MMV2_MYCA9  EFPKGGIHNWTYWGNQLRAMKADLVGYLTKPGTAAV 331
                     ::  .* *.* *** **.*** ** . *
```

Supplementary Figure S1

Alignment of MTB and NTM Ag85 protein sequences.

A Clustal Omega multiple sequence alignment [citation] of Ag85 protein sequences from various *Mycobacterium* strains is shown. The MTB strains include P9WQP1 (*M. tuberculosis*), P0C2T2 (*M. bovis* ATCC), and A1KJU9 (*M. bovis* BCG). The NTM strains include Q49575 (*M. intracellulare*), P21160 (*M. kansasii*), Q06947 (*M. avium*), and B1MMV2 (*M. abscessus*). The alignment highlights a unique amino acid sequence (TQQIPK) at positions 234-239 (red underline) present in the MTB strains but absent in the NTM strains.

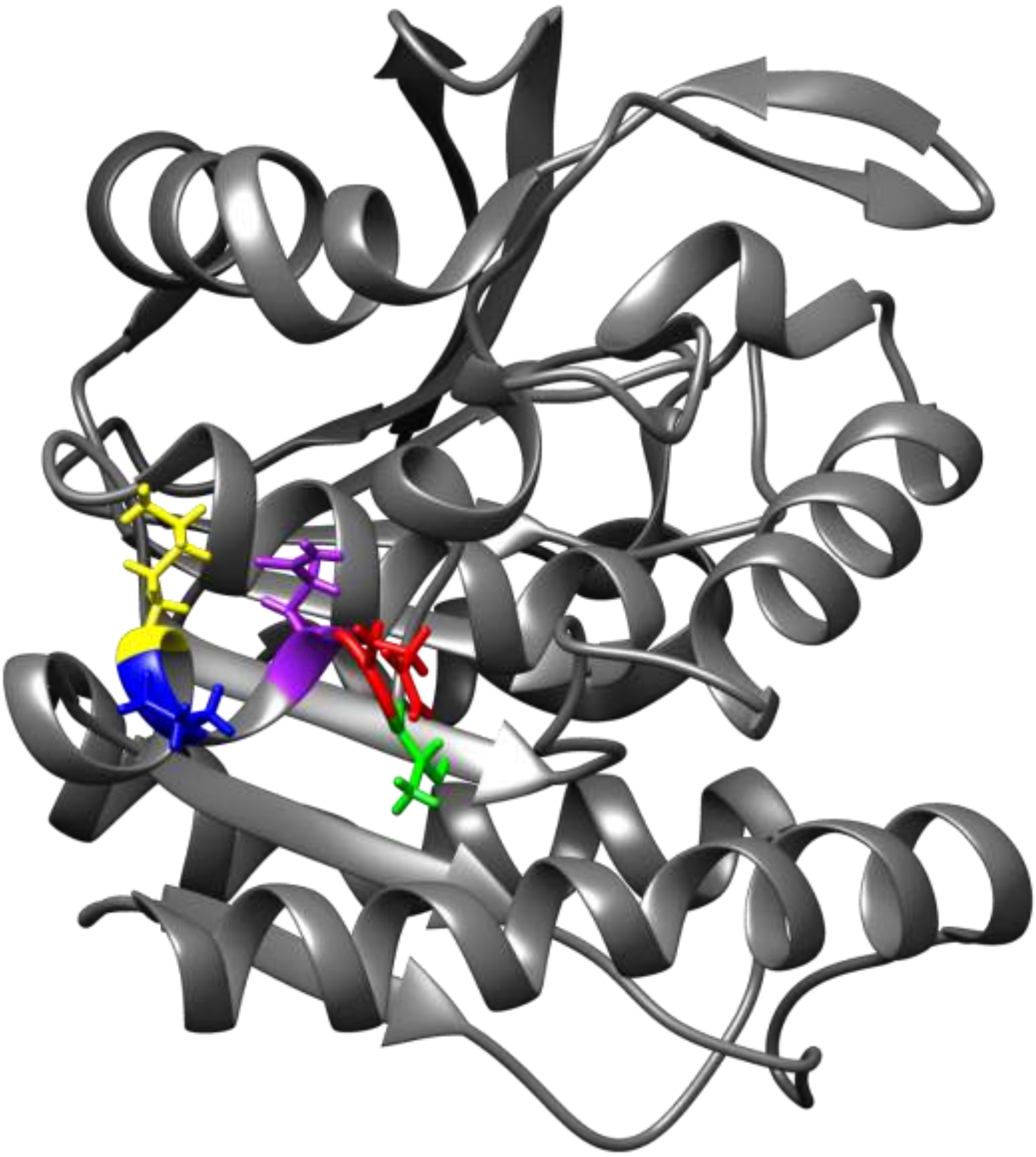

Supplementary Figure S2
Prediction of MTB-Ag85 (PDB 1F0N) epitope by BCEP.
A surface-accessible epitope including T^{234} (green), Q^{235} (red), Q^{236} (purple), P^{238} (blue), and K^{239} (yellow) is depicted. The conserved I^{236} was buried within the structure.
